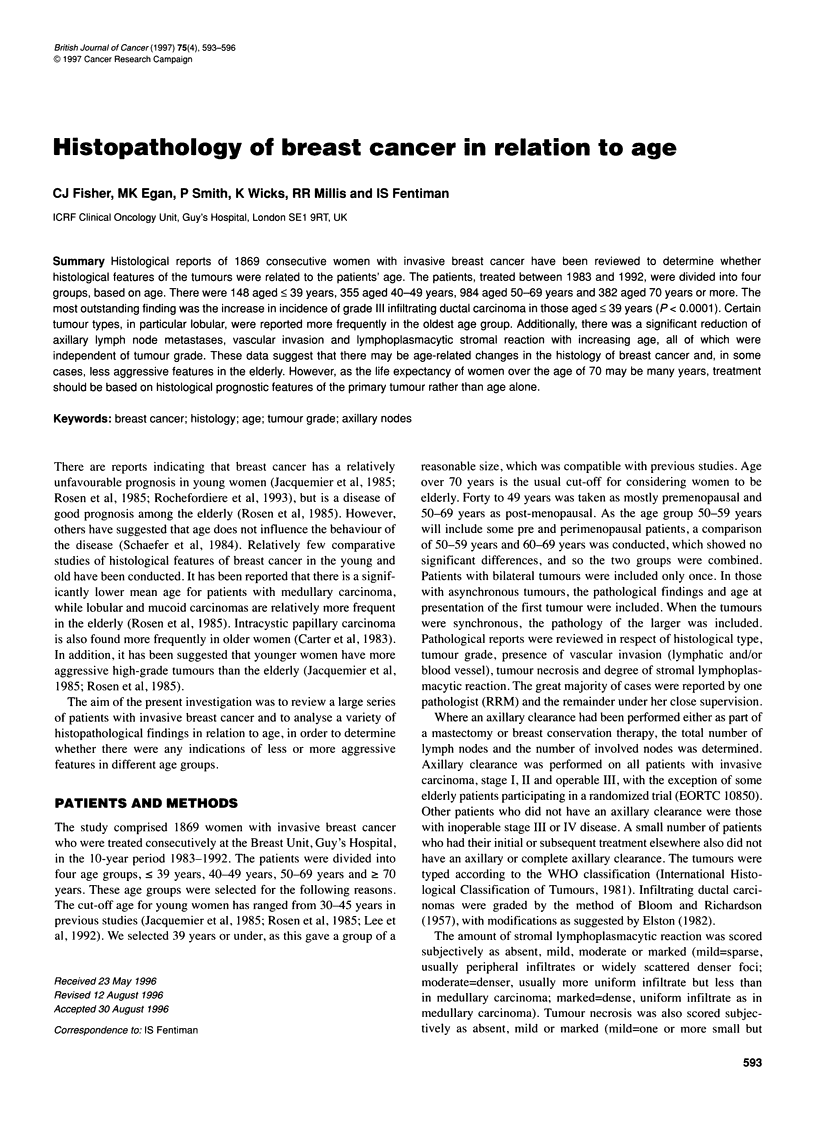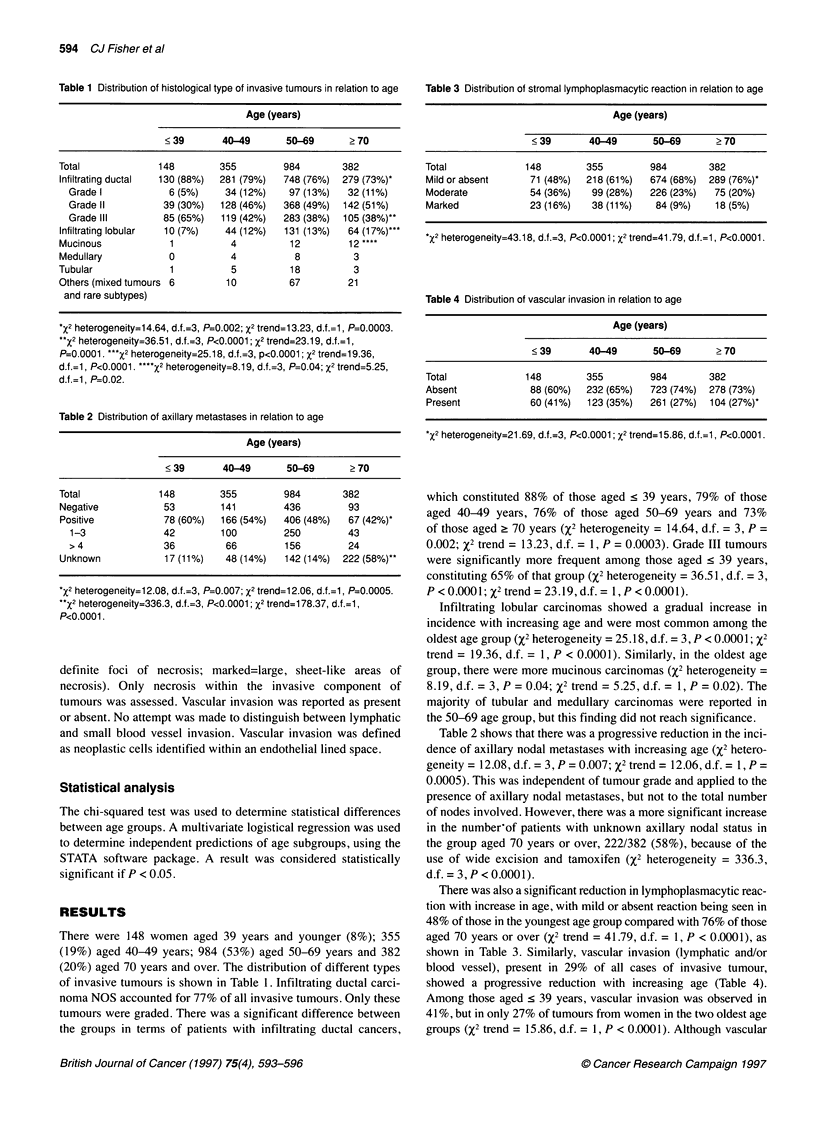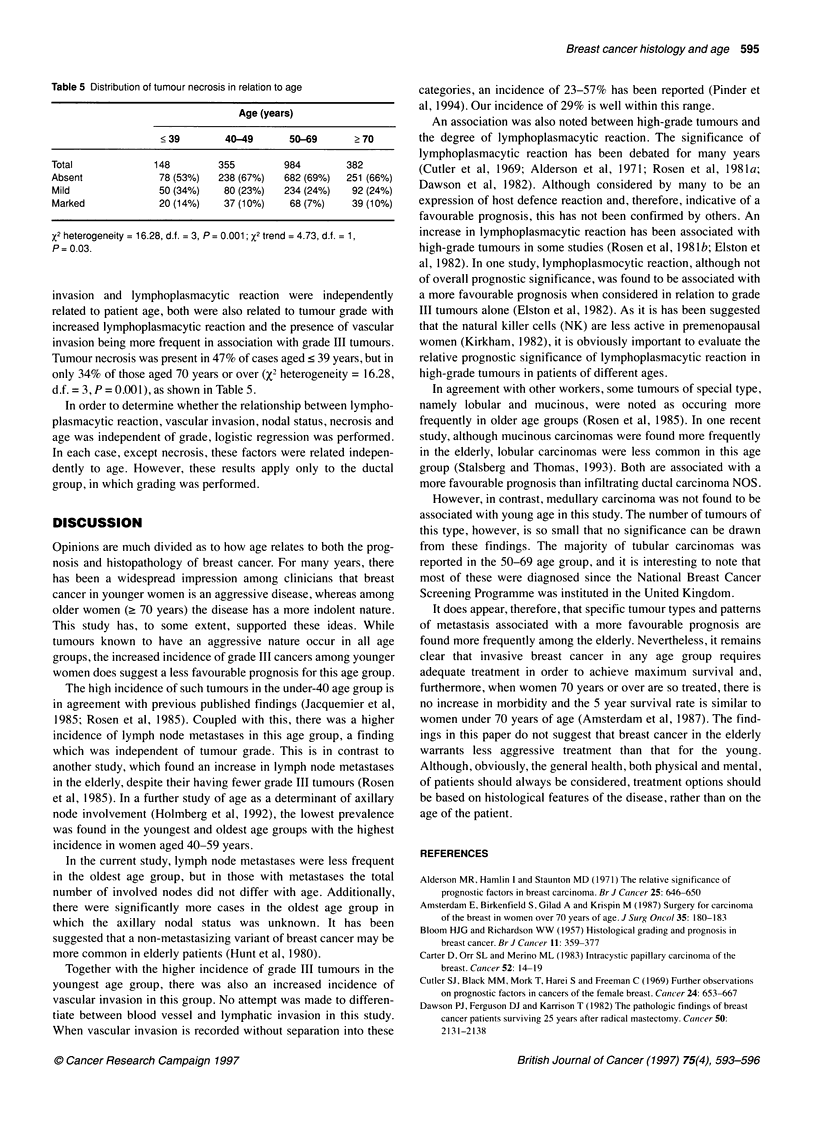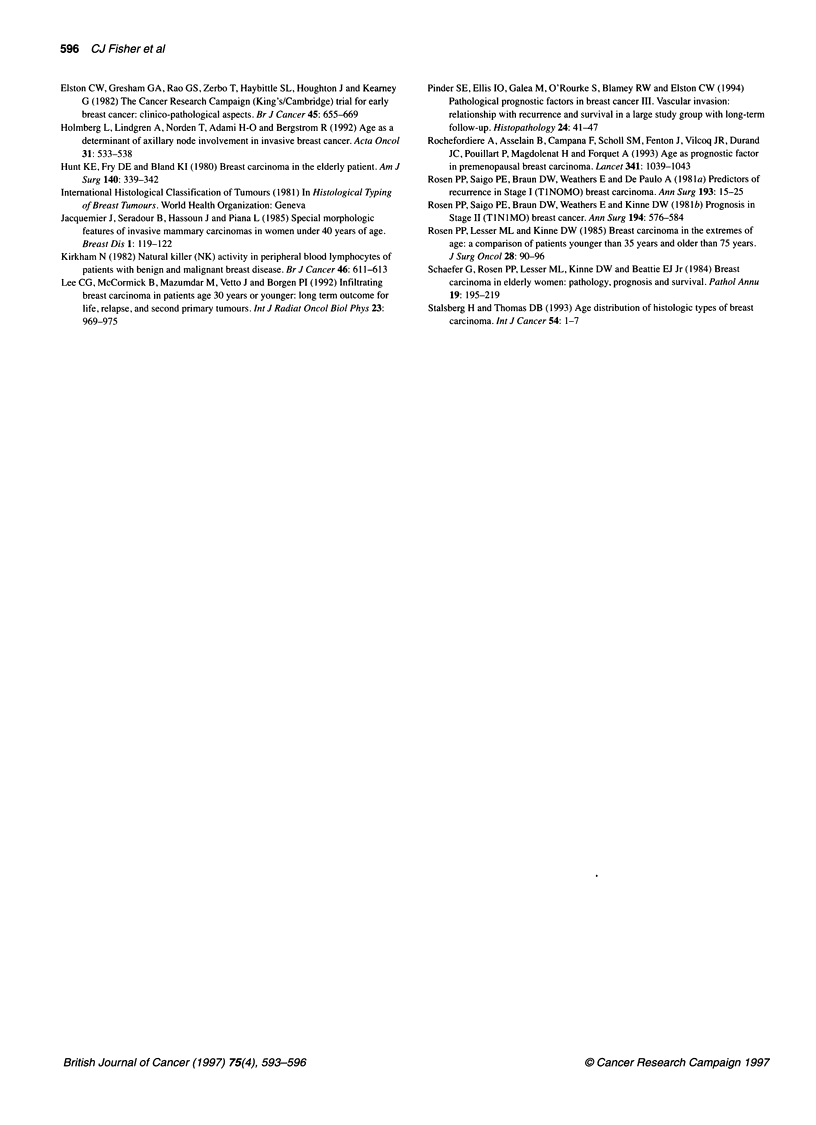# Histopathology of breast cancer in relation to age.

**DOI:** 10.1038/bjc.1997.103

**Published:** 1997

**Authors:** C. J. Fisher, M. K. Egan, P. Smith, K. Wicks, R. R. Millis, I. S. Fentiman

**Affiliations:** ICRF Clinical Oncology Unit, Guy's Hospital, London, UK.

## Abstract

Histological reports of 1869 consecutive women with invasive breast cancer have been reviewed to determine whether histological features of the tumour's were related to the patients' age. The patients, treated between 1983 and 1992, were divided into four groups, based on age. There were 148 aged < or = 39 years, 355 aged 40-49 years, 984 aged 50-69 years and 382 aged 70 years or more. The most outstanding finding was the increase in incidence of grade III infiltrating ductal carcinoma in those aged < or = 39 years (P < 0.0001). Certain tumour types, in particular lobular, were reported more frequently in the oldest age group. Additionally, there was a significant reduction of axillary lymph node metastases, vascular invasion and lymphoplasmacytic stromal reaction with increasing age, all of which were independent of tumour grade. These data suggest that there may be age-related changes in the histology of breast cancer and, in some cases, less aggressive features in the elderly. However, as the life expectancy of women over the age of 70 may be many years, treatment should be based on histological prognostic features of the primary tumour rather than age alone.


*British Journal of Cancer* (1997) **75**(4), 593–596
© 1997 Cancer Research Campaign

### Histopathology of breast cancer in relation to age

**CJ Fisher, MK Egan, P Smith, K Wicks, RR Millis and IS Fentiman**

ICRF Clinical Oncology Unit, Guy's Hospital, London SE1 9RT, UK

**Summary** Histological reports of 1869 consecutive women with invasive breast cancer have been reviewed to determine whether histological features of the tumours were related to the patients' age. The patients, treated between 1983 and 1992, were divided into four groups, based on age. There were 148 aged ≤ 39 years, 355 aged 40–49 years, 984 aged 50–69 years and 382 aged 70 years or more. The most outstanding finding was the increase in incidence of grade III infiltrating ductal carcinoma in those aged ≤ 39 years ($P < 0.0001$). Certain tumour types, in particular lobular, were reported more frequently in the oldest age group. Additionally, there was a significant reduction of axillary lymph node metastases, vascular invasion and lymphoplasmacytic stromal reaction with increasing age, all of which were independent of tumour grade. These data suggest that there may be age-related changes in the histology of breast cancer and, in some cases, less aggressive features in the elderly. However, as the life expectancy of women over the age of 70 may be many years, treatment should be based on histological prognostic features of the primary tumour rather than age alone.

**Keywords:** breast cancer; histology; age; tumour grade; axillary nodes

There are reports indicating that breast cancer has a relatively unfavourable prognosis in young women (Jacquemier et al, 1985; Rosen et al, 1985; Rochefordiere et al, 1993), but is a disease of good prognosis among the elderly (Rosen et al, 1985). However, others have suggested that age does not influence the behaviour of the disease (Schaefer et al, 1984). Relatively few comparative studies of histological features of breast cancer in the young and old have been conducted. It has been reported that there is a significantly lower mean age for patients with medullary carcinoma, while lobular and mucoid carcinomas are relatively more frequent in the elderly (Rosen et al, 1985). Intracystic papillary carcinoma is also found more frequently in older women (Carter et al, 1983). In addition, it has been suggested that younger women have more aggressive high-grade tumours than the elderly (Jacquemier et al, 1985; Rosen et al, 1985).

The aim of the present investigation was to review a large series of patients with invasive breast cancer and to analyse a variety of histopathological findings in relation to age, in order to determine whether there were any indications of less or more aggressive features in different age groups.

### PATIENTS AND METHODS

The study comprised 1869 women with invasive breast cancer who were treated consecutively at the Breast Unit, Guy's Hospital, in the 10-year period 1983–1992. The patients were divided into four age groups, ≤ 39 years, 40–49 years, 50–69 years and ≥ 70 years. These age groups were selected for the following reasons. The cut-off age for young women has ranged from 30–45 years in previous studies (Jacquemier et al, 1985; Rosen et al, 1985; Lee et al, 1992). We selected 39 years or under, as this gave a group of a reasonable size, which was compatible with previous studies. Age over 70 years is the usual cut-off for considering women to be elderly. Forty to 49 years was taken as mostly premenopausal and 50–69 years as post-menopausal. As the age group 50–59 years will include some pre and perimenopausal patients, a comparison of 50–59 years and 60–69 years was conducted, which showed no significant differences, and so the two groups were combined. Patients with bilateral tumours were included only once. In those with asynchronous tumours, the pathological findings and age at presentation of the first tumour were included. When the tumours were synchronous, the pathology of the larger was included. Pathological reports were reviewed in respect of histological type, tumour grade, presence of vascular invasion (lymphatic and/or blood vessel), tumour necrosis and degree of stromal lymphoplasmacytic reaction. The great majority of cases were reported by one pathologist (RRM) and the remainder under her close supervision.

Where an axillary clearance had been performed either as part of a mastectomy or breast conservation therapy, the total number of lymph nodes and the number of involved nodes was determined. Axillary clearance was performed on all patients with invasive carcinoma, stage I, II and operable III, with the exception of some elderly patients participating in a randomized trial (EORTC 10850). Other patients who did not have an axillary clearance were those with inoperable stage III or IV disease. A small number of patients who had their initial or subsequent treatment elsewhere also did not have an axillary or complete axillary clearance. The tumours were typed according to the WHO classification (International Histological Classification of Tumours, 1981). Infiltrating ductal carcinomas were graded by the method of Bloom and Richardson (1957), with modifications as suggested by Elston (1982).

The amount of stromal lymphoplasmacytic reaction was scored subjectively as absent, mild, moderate or marked (mild=sparse, usually peripheral infiltrates or widely scattered denser foci; moderate=denser, usually more uniform infiltrate but less than in medullary carcinoma; marked=dense, uniform infiltrate as in medullary carcinoma). Tumour necrosis was also scored subjectively as absent, mild or marked (mild=one or more small but

*Received 23 May 1996*
*Revised 12 August 1996*
*Accepted 30 August 1996*

*Correspondence to:* IS Fentiman

**Table 1** Distribution of histological type of invasive tumours in relation to age

| | Age (years) | | | |
|---|---|---|---|---|
| | ≤ 39 | 40–49 | 50–69 | ≥ 70 |
| Total | 148 | 355 | 984 | 382 |
| Infiltrating ductal | 130 (88%) | 281 (79%) | 748 (76%) | 279 (73%)* |
| Grade I | 6 (5%) | 34 (12%) | 97 (13%) | 32 (11%) |
| Grade II | 39 (30%) | 128 (46%) | 368 (49%) | 142 (51%) |
| Grade III | 85 (65%) | 119 (42%) | 283 (38%) | 105 (38%)** |
| Infiltrating lobular | 10 (7%) | 44 (12%) | 131 (13%) | 64 (17%)*** |
| Mucinous | 1 | 4 | 12 | 12 **** |
| Medullary | 0 | 4 | 8 | 3 |
| Tubular | 1 | 5 | 18 | 3 |
| Others (mixed tumours and rare subtypes) | 6 | 10 | 67 | 21 |

*$\chi^2$ heterogeneity=14.64, d.f.=3, *P*=0.002; $\chi^2$ trend=13.23, d.f.=1, *P*=0.0003. **$\chi^2$ heterogeneity=36.51, d.f.=3, *P*<0.0001; $\chi^2$ trend=23.19, d.f.=1, *P*=0.0001. ***$\chi^2$ heterogeneity=25.18, d.f.=3, p<0.0001; $\chi^2$ trend=19.36, d.f.=1, *P*<0.0001. ****$\chi^2$ heterogeneity=8.19, d.f.=3, *P*=0.04; $\chi^2$ trend=5.25, d.f.=1, *P*=0.02.

**Table 2** Distribution of axillary metastases in relation to age

| | Age (years) | | | |
|---|---|---|---|---|
| | ≤ 39 | 40–49 | 50–69 | ≥ 70 |
| Total | 148 | 355 | 984 | 382 |
| Negative | 53 | 141 | 436 | 93 |
| Positive | 78 (60%) | 166 (54%) | 406 (48%) | 67 (42%)* |
| 1–3 | 42 | 100 | 250 | 43 |
| > 4 | 36 | 66 | 156 | 24 |
| Unknown | 17 (11%) | 48 (14%) | 142 (14%) | 222 (58%)** |

*$\chi^2$ heterogeneity=12.08, d.f.=3, *P*=0.007; $\chi^2$ trend=12.06, d.f.=1, *P*=0.0005. **$\chi^2$ heterogeneity=336.3, d.f.=3, *P*<0.0001; $\chi^2$ trend=178.37, d.f.=1, *P*<0.0001.

**Table 3** Distribution of stromal lymphoplasmacytic reaction in relation to age

| | Age (years) | | | |
|---|---|---|---|---|
| | ≤ 39 | 40–49 | 50–69 | ≥ 70 |
| Total | 148 | 355 | 984 | 382 |
| Mild or absent | 71 (48%) | 218 (61%) | 674 (68%) | 289 (76%)* |
| Moderate | 54 (36%) | 99 (28%) | 226 (23%) | 75 (20%) |
| Marked | 23 (16%) | 38 (11%) | 84 (9%) | 18 (5%) |

*$\chi^2$ heterogeneity=43.18, d.f.=3, *P*<0.0001; $\chi^2$ trend=41.79, d.f.=1, *P*<0.0001.

**Table 4** Distribution of vascular invasion in relation to age

| | Age (years) | | | |
|---|---|---|---|---|
| | ≤ 39 | 40–49 | 50–69 | ≥ 70 |
| Total | 148 | 355 | 984 | 382 |
| Absent | 88 (60%) | 232 (65%) | 723 (74%) | 278 (73%) |
| Present | 60 (41%) | 123 (35%) | 261 (27%) | 104 (27%)* |

*$\chi^2$ heterogeneity=21.69, d.f.=3, *P*<0.0001; $\chi^2$ trend=15.86, d.f.=1, *P*<0.0001.

definite foci of necrosis; marked=large, sheet-like areas of necrosis). Only necrosis within the invasive component of tumours was assessed. Vascular invasion was reported as present or absent. No attempt was made to distinguish between lymphatic and small blood vessel invasion. Vascular invasion was defined as neoplastic cells identified within an endothelial lined space.

### Statistical analysis

The chi-squared test was used to determine statistical differences between age groups. A multivariate logistical regression was used to determine independent predictions of age subgroups, using the STATA software package. A result was considered statistically significant if $P < 0.05$.

### RESULTS

There were 148 women aged 39 years and younger (8%); 355 (19%) aged 40–49 years; 984 (53%) aged 50–69 years and 382 (20%) aged 70 years and over. The distribution of different types of invasive tumours is shown in Table 1. Infiltrating ductal carcinoma NOS accounted for 77% of all invasive tumours. Only these tumours were graded. There was a significant difference between the groups in terms of patients with infiltrating ductal cancers, which constituted 88% of those aged ≤ 39 years, 79% of those aged 40–49 years, 76% of those aged 50–69 years and 73% of those aged ≥ 70 years ($\chi^2$ heterogeneity = 14.64, d.f. = 3, $P$ = 0.002; $\chi^2$ trend = 13.23, d.f. = 1, $P$ = 0.0003). Grade III tumours were significantly more frequent among those aged ≤ 39 years, constituting 65% of that group ($\chi^2$ heterogeneity = 36.51, d.f. = 3, $P < 0.0001$; $\chi^2$ trend = 23.19, d.f. = 1, $P < 0.0001$).

Infiltrating lobular carcinomas showed a gradual increase in incidence with increasing age and were most common among the oldest age group ($\chi^2$ heterogeneity = 25.18, d.f. = 3, $P < 0.0001$; $\chi^2$ trend = 19.36, d.f. = 1, $P < 0.0001$). Similarly, in the oldest age group, there were more mucinous carcinomas ($\chi^2$ heterogeneity = 8.19, d.f. = 3, $P$ = 0.04; $\chi^2$ trend = 5.25, d.f. = 1, $P$ = 0.02). The majority of tubular and medullary carcinomas were reported in the 50–69 age group, but this finding did not reach significance.

Table 2 shows that there was a progressive reduction in the incidence of axillary nodal metastases with increasing age ($\chi^2$ heterogeneity = 12.08, d.f. = 3, $P$ = 0.007; $\chi^2$ trend = 12.06, d.f. = 1, $P$ = 0.0005). This was independent of tumour grade and applied to the presence of axillary nodal metastases, but not to the total number of nodes involved. However, there was a more significant increase in the number·of patients with unknown axillary nodal status in the group aged 70 years or over, 222/382 (58%), because of the use of wide excision and tamoxifen ($\chi^2$ heterogeneity = 336.3, d.f. = 3, $P < 0.0001$).

There was also a significant reduction in lymphoplasmacytic reaction with increase in age, with mild or absent reaction being seen in 48% of those in the youngest age group compared with 76% of those aged 70 years or over ($\chi^2$ trend = 41.79, d.f. = 1, $P < 0.0001$), as shown in Table 3. Similarly, vascular invasion (lymphatic and/or blood vessel), present in 29% of all cases of invasive tumour, showed a progressive reduction with increasing age (Table 4). Among those aged ≤ 39 years, vascular invasion was observed in 41%, but in only 27% of tumours from women in the two oldest age groups ($\chi^2$ trend = 15.86, d.f. = 1, $P < 0.0001$). Although vascular

© Cancer Research Campaign 1997

**Table 5** Distribution of tumour necrosis in relation to age

| | Age (years) | | | |
|---|---|---|---|---|
| | ≤ 39 | 40–49 | 50–69 | ≥ 70 |
| Total | 148 | 355 | 984 | 382 |
| Absent | 78 (53%) | 238 (67%) | 682 (69%) | 251 (66%) |
| Mild | 50 (34%) | 80 (23%) | 234 (24%) | 92 (24%) |
| Marked | 20 (14%) | 37 (10%) | 68 (7%) | 39 (10%) |

$\chi^2$ heterogeneity = 16.28, d.f. = 3, $P = 0.001$; $\chi^2$ trend = 4.73, d.f. = 1, $P = 0.03$.

invasion and lymphoplasmacytic reaction were independently related to patient age, both were also related to tumour grade with increased lymphoplasmacytic reaction and the presence of vascular invasion being more frequent in association with grade III tumours. Tumour necrosis was present in 47% of cases aged ≤ 39 years, but in only 34% of those aged 70 years or over ($\chi^2$ heterogeneity = 16.28, d.f. = 3, $P = 0.001$), as shown in Table 5.

In order to determine whether the relationship between lymphoplasmacytic reaction, vascular invasion, nodal status, necrosis and age was independent of grade, logistic regression was performed. In each case, except necrosis, these factors were related independently to age. However, these results apply only to the ductal group, in which grading was performed.

### DISCUSSION

Opinions are much divided as to how age relates to both the prognosis and histopathology of breast cancer. For many years, there has been a widespread impression among clinicians that breast cancer in younger women is an aggressive disease, whereas among older women (≥ 70 years) the disease has a more indolent nature. This study has, to some extent, supported these ideas. While tumours known to have an aggressive nature occur in all age groups, the increased incidence of grade III cancers among younger women does suggest a less favourable prognosis for this age group.

The high incidence of such tumours in the under-40 age group is in agreement with previous published findings (Jacquemier et al, 1985; Rosen et al, 1985). Coupled with this, there was a higher incidence of lymph node metastases in this age group, a finding which was independent of tumour grade. This is in contrast to another study, which found an increase in lymph node metastases in the elderly, despite their having fewer grade III tumours (Rosen et al, 1985). In a further study of age as a determinant of axillary node involvement (Holmberg et al, 1992), the lowest prevalence was found in the youngest and oldest age groups with the highest incidence in women aged 40–59 years.

In the current study, lymph node metastases were less frequent in the oldest age group, but in those with metastases the total number of involved nodes did not differ with age. Additionally, there were significantly more cases in the oldest age group in which the axillary nodal status was unknown. It has been suggested that a non-metastasizing variant of breast cancer may be more common in elderly patients (Hunt et al, 1980).

Together with the higher incidence of grade III tumours in the youngest age group, there was also an increased incidence of vascular invasion in this group. No attempt was made to differentiate between blood vessel and lymphatic invasion in this study. When vascular invasion is recorded without separation into these categories, an incidence of 23–57% has been reported (Pinder et al, 1994). Our incidence of 29% is well within this range.

An association was also noted between high-grade tumours and the degree of lymphoplasmacytic reaction. The significance of lymphoplasmacytic reaction has been debated for many years (Cutler et al, 1969; Alderson et al, 1971; Rosen et al, 1981*a*; Dawson et al, 1982). Although considered by many to be an expression of host defence reaction and, therefore, indicative of a favourable prognosis, this has not been confirmed by others. An increase in lymphoplasmacytic reaction has been associated with high-grade tumours in some studies (Rosen et al, 1981*b*; Elston et al, 1982). In one study, lymphoplasmocytic reaction, although not of overall prognostic significance, was found to be associated with a more favourable prognosis when considered in relation to grade III tumours alone (Elston et al, 1982). As it is has been suggested that the natural killer cells (NK) are less active in premenopausal women (Kirkham, 1982), it is obviously important to evaluate the relative prognostic significance of lymphoplasmacytic reaction in high-grade tumours in patients of different ages.

In agreement with other workers, some tumours of special type, namely lobular and mucinous, were noted as occuring more frequently in older age groups (Rosen et al, 1985). In one recent study, although mucinous carcinomas were found more frequently in the elderly, lobular carcinomas were less common in this age group (Stalsberg and Thomas, 1993). Both are associated with a more favourable prognosis than infiltrating ductal carcinoma NOS.

However, in contrast, medullary carcinoma was not found to be associated with young age in this study. The number of tumours of this type, however, is so small that no significance can be drawn from these findings. The majority of tubular carcinomas was reported in the 50–69 age group, and it is interesting to note that most of these were diagnosed since the National Breast Cancer Screening Programme was instituted in the United Kingdom.

It does appear, therefore, that specific tumour types and patterns of metastasis associated with a more favourable prognosis are found more frequently among the elderly. Nevertheless, it remains clear that invasive breast cancer in any age group requires adequate treatment in order to achieve maximum survival and, furthermore, when women 70 years or over are so treated, there is no increase in morbidity and the 5 year survival rate is similar to women under 70 years of age (Amsterdam et al, 1987). The findings in this paper do not suggest that breast cancer in the elderly warrants less aggressive treatment than that for the young. Although, obviously, the general health, both physical and mental, of patients should always be considered, treatment options should be based on histological features of the disease, rather than on the age of the patient.

### REFERENCES

Alderson MR, Hamlin I and Staunton MD (1971) The relative significance of prognostic factors in breast carcinoma. *Br J Cancer* **25**: 646–650

Amsterdam E, Birkenfield S, Gilad A and Krispin M (1987) Surgery for carcinoma of the breast in women over 70 years of age. *J Surg Oncol* **35**: 180–183

Bloom HJG and Richardson WW (1957) Histological grading and prognosis in breast cancer. *Br J Cancer* **11**: 359–377

Carter D, Orr SL and Merino ML (1983) Intracystic papillary carcinoma of the breast. *Cancer* **52**: 14–19

Cutler SJ, Black MM, Mork T, Harei S and Freeman C (1969) Further observations on prognostic factors in cancers of the female breast. *Cancer* **24**: 653–667

Dawson PJ, Ferguson DJ and Karrison T (1982) The pathologic findings of breast cancer patients surviving 25 years after radical mastectomy. *Cancer* **50**: 2131–2138

© Cancer Research Campaign 1997

Elston CW, Gresham GA, Rao GS, Zerbo T, Haybittle SL, Houghton J and Kearney G (1982) The Cancer Research Campaign (King's/Cambridge) trial for early breast cancer: clinico-pathological aspects. *Br J Cancer* **45**: 655–669

Holmberg L, Lindgren A, Norden T, Adami H-O and Bergstrom R (1992) Age as a determinant of axillary node involvement in invasive breast cancer. *Acta Oncol* **31**: 533–538

Hunt KE, Fry DE and Bland KI (1980) Breast carcinoma in the elderly patient. *Am J Surg* **140**: 339–342

International Histological Classification of Tumours (1981) In *Histological Typing of Breast Tumours*. World Health Organization: Geneva

Jacquemier J, Seradour B, Hassoun J and Piana L (1985) Special morphologic features of invasive mammary carcinomas in women under 40 years of age. *Breast Dis* **1**: 119–122

Kirkham N (1982) Natural killer (NK) activity in peripheral blood lymphocytes of patients with benign and malignant breast disease. *Br J Cancer* **46**: 611–613

Lee CG, McCormick B, Mazumdar M, Vetto J and Borgen PI (1992) Infiltrating breast carcinoma in patients age 30 years or younger: long term outcome for life, relapse, and second primary tumours. *Int J Radiat Oncol Biol Phys* **23**: 969–975

Pinder SE, Ellis IO, Galea M, O'Rourke S, Blamey RW and Elston CW (1994) Pathological prognostic factors in breast cancer III. Vascular invasion: relationship with recurrence and survival in a large study group with long-term follow-up. *Histopathology* **24**: 41–47

Rochefordiere A, Asselain B, Campana F, Scholl SM, Fenton J, Vilcoq JR, Durand JC, Pouillart P, Magdolenat H and Forquet A (1993) Age as prognostic factor in premenopausal breast carcinoma. *Lancet* **341**: 1039–1043

Rosen PP, Saigo PE, Braun DW, Weathers E and De Paulo A (1981*a*) Predictors of recurrence in Stage I (T1NOMO) breast carcinoma. *Ann Surg* **193**: 15–25

Rosen PP, Saigo PE, Braun DW, Weathers E and Kinne DW (1981*b*) Prognosis in Stage II (T1N1MO) breast cancer. *Ann Surg* **194**: 576–584

Rosen PP, Lesser ML and Kinne DW (1985) Breast carcinoma in the extremes of age: a comparison of patients younger than 35 years and older than 75 years. *J Surg Oncol* **28**: 90–96

Schaefer G, Rosen PP, Lesser ML, Kinne DW and Beattie EJ Jr (1984) Breast carcinoma in elderly women: pathology, prognosis and survival. *Pathol Annu* **19**: 195–219

Stalsberg H and Thomas DB (1993) Age distribution of histologic types of breast carcinoma. *Int J Cancer* **54**: 1–7

© Cancer Research Campaign 1997